# Vibration responses characteristics of a *Ginkgo biloba* tree excited under harmonic excitation

**DOI:** 10.1371/journal.pone.0256492

**Published:** 2021-08-20

**Authors:** Huan Lin, Leihou Sun

**Affiliations:** Department of Intelligent Equipment, Changzhou College of Information Technology, Changzhou, Jiangsu, China; US Department of Agriculture, UNITED STATES

## Abstract

The most effective method of the fruit harvesting is the mechanical harvest. The frequency spectrum of different testing positions on a *Ginkgo biloba* tree under the impact excitation was tested in the laboratory. The acceleration responses under the harmonic excitation were measured at the frequency of the peak and trough points in the frequency spectrum curves. Results of this research indicate that the frequency spectrum presented the consistency on the same branch but distinction among different branches. There was a correspondence between the frequency spectrum characteristics and the vibration responses. The vibration responses could be strengthened at the resonant frequency. Merely, the acceleration responses at low frequency were very weak. At higher frequency, the vibration responses were strong but presented different characteristics among different branches. The acceleration response on the trunk was always the weakest. On the same branch, the dynamic responses presented the similar characteristics and the acceleration amplitude increased gradually as the testing position was located away from the excitation point on the trunk. Among different branches, the strongest dynamic response appeared at different frequencies. Our results indicate that it was difficult to induce the strong vibration response of all the branches at the single frequency during the practical mechanical harvesting of fruits.

## 1. Introduction

At present, mechanical harvest is the most effective method for the fruit harvesting [1–3]. Mechanical harvesters usually use the shaking or vibratory methods, such as air shaking, trunk shaking, limb shaking and canopy shaking [4,5]. The basic principle of vibratory harvesting is to transmit vibration energy to fruiting branches and then convert the energy into the traction force on fruit-stems. Fruits removal will occur when the traction force exceeds the tensile force of fruit-stems [6–8].

Studies were carried out to improve the removal efficiency of fruits. Torregrosa et al [9] used trunk and hand-held shakers to harvest several varieties of oranges and mandarins. Results showed that the tractor shaker was more effective (72% detachment) than hand-held shakers (57% detachment). Tests and analyses reflected that excitation amplitude, frequency, duration and position affected the removal efficiency and the damage of fruits [10–12]. Loghavi et al [13] found that shaking the limbs at 80 mm amplitude and 10 Hz frequency with 98.5% lime fruit detachment was the most suitable combination. Erdoğan et al [14] discovered that the inertia type limb shaker should be operated within the range of 40 mm amplitude and 15Hz frequency to obtain maximum fruit removal with minimum vibration and reactive force. Mateev et al [15] established a probabilistic model for describing the vibratory fruit removal under different specified harvesting conditions and then validated the model precision. Harvesting robots were also studied to conduct basic experiments of the individual fruit or a targeted group of fruit [16,17].

The vibration response and energy transmission efficiency of the fruit trees were also researched. Du et al [18] studied the dynamic response of UFO (upright fruiting offshoots) cherry trees under the forced vibrations, and found that when the limb was excited at the low end near the trunk, the acceleration response of wood reached the maximum in the middle portion of both the trunk (horizontal) and the offshoots (vertical) at their resonant frequencies. Du et al [19] studied the dynamic characteristics of dwarf Chinese hickory trees under impact excitation and found that the variation of the dynamic response along the testing tree was greatly related to the crotch angle and the branch chain configuration. He et al [20] also studied the energy transmission of excited and neighboring limbs using a mechanical shaker in the “Y” trellised cherry orchard. Results revealed that the majority of energy (approximately 85% at 14 Hz shaking) was transmitted to the excited limbs. The variation of the energy distribution along a limb excited at different positions brings the possibility of obtaining different fruit removal efficiencies. The energy equation to the crank slider and eccentric block vibratory harvesters was studied [21–23]. The vibration system including the dynamic damping coefficient, elastic coefficient and equivalent mass was established. The relationship between the excitation height and frequency was also concluded.

Tree generally grows into the branch-on-branch structure and the limb vibration induced by the external force is often unevenly distributed on different parts of the tree [24]. Therefore, a better understanding of the behavior and dynamic characteristics of plants under forced vibration would help in designing mechanical harvesters [25]. Castro-García, et al [26] tested the modal parameters of olive trees and found that they were determined by the mass distribution, stiffness, and morphology. A series of experimental modal analysis was performed by using the stepping sinusoidal excitation, one-position excitation and multi-position acceleration response method. The wide distributions of data were shown in the damping ratio, modal mass and modal stiffness of the blackcurrant branches [27]. Rodriguez et al [28] analyzed the case of an idealized sympodial tree to understand the multimodal dynamics of trees and to quantitatively explain the role of geometry on their dynamical characteristics. Rodriguez et al [29] studied the vibration modes of the tree and validated a previous analytical approach that predicted the organization of modal frequencies as a function of two allometry parameters. Moore et al [30] tested the natural frequency and damping ratio of trees, then pointed out that it might not be appropriate to treat branches as lumped masses rather than individual cantilevers attached to the main stem.

During the vibratory harvest, the traction force on fruit-stems has the positive correlation with the acceleration in spatial directions. Whereas, Du et al [30,31] only studied the axis parallel to the direction of excitation and the maximum acceleration, which couldn’t completely reflect the response characteristics of trees. Although the spectral analysis method was the conventional method during the signal analysis, the frequency response characteristics between the anisotropic live trees and the isotropous metal components had no comparability. Therefore, the frequency spectrum characteristics and the corresponding vibration responses in spatial directions on a *Ginkgo biloba* tree were determined. The objective of the current work was to find the relationship between the frequency spectrum characteristics and the vibration responses.

## 2. Materials and methods

### 2.1 Experimental materials and distribution of testing positions

**Fig 1:**
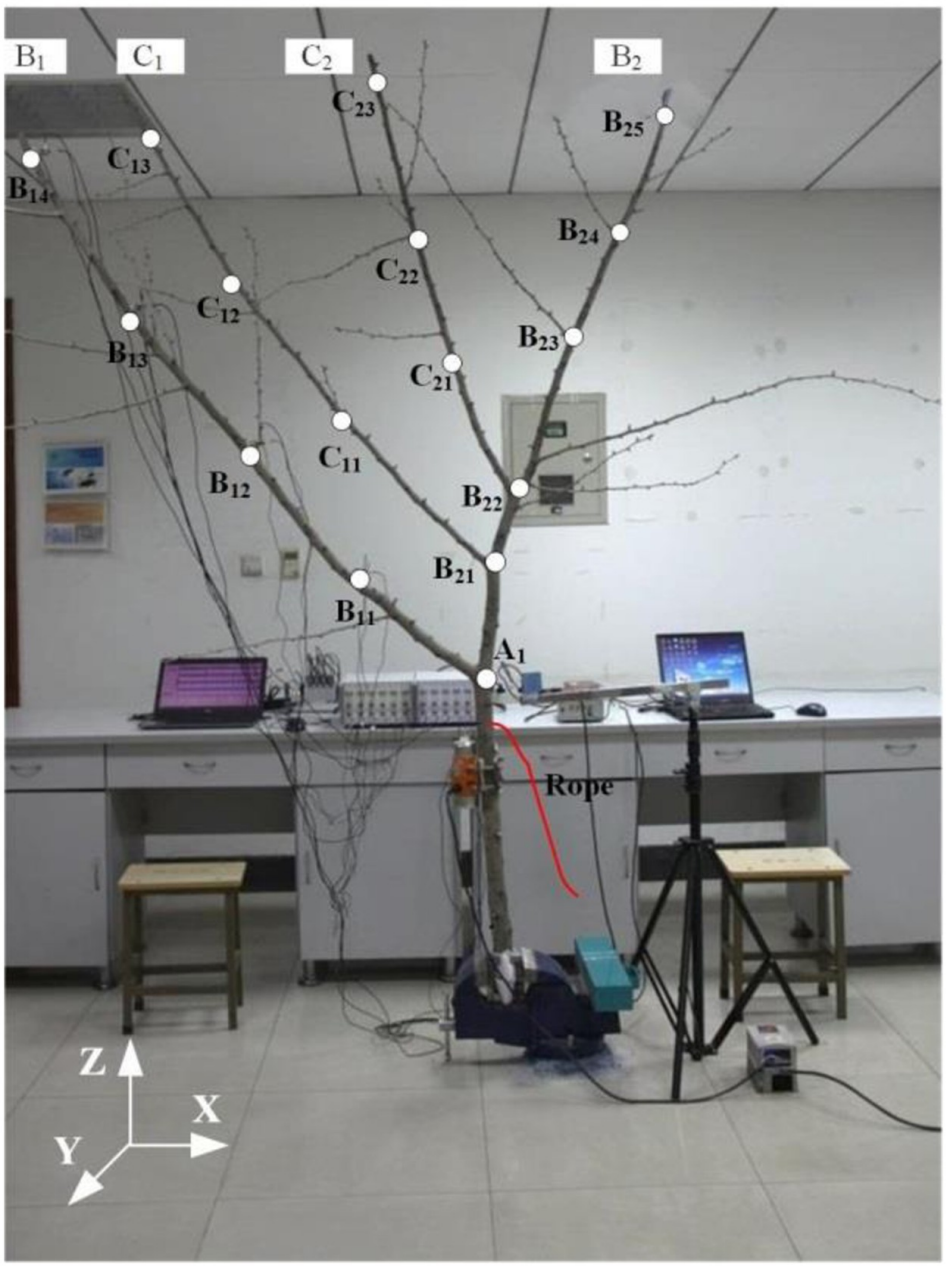
Diagram of tree specimen and data acquisition setup.

There was distinction among the physical and kinetic characteristics of different *Ginkgo biloba* trees. By pre-experiment, we have discovered that the fundamental frequency of *Ginkgo biloba* tree was low and strong acceleration response couldn’t be induced by the fundamental frequency [32]. In order to completely analyze the relationship between the frequency spectrum characteristics and the vibration responses of different branches, one specimen was selected for the experiments and analysis. A 4-year-old Chinese *Ginkgo biloba* tree was selected as the experimental specimen from Nanjing Forestry University (Nanjing, Jiangsu Province, China, 32.1°N, 118.8°E). As shown in Fig 1, it mainly consisted of a trunk A from A_0_ to A_1_, two first branches B_1_, B_2_ originating from the trunk and two second branches C_1_, C_2_ originating from branch B_2_. All the branches were almost located in the same perpendicular plane.

The root and leaves were removed and the total mass of this tree specimen was 4.46 kg. During the experiment, the segment below position A_0_ was fixed vertically using clamps to ground. Testing positions A_1_, B_21_, B_22_ were located at three crotch nodes and the other testing positions were averagely arranged according to the length of each branch. Every testing position was marked by a white circle. The major branch indexes and their geometric parameters were listed in Table 1. The experiments were conducted immediately after the tree specimen was delivered to the laboratory (March 16–18, 2020). The relative humidity of the laboratory was maintained at 60% and the temperature was controlled at 25°C.

**Table 1:** Dimension parameters of a *Ginkgo biloba* tree illustrated in Fig 1.

Branch rank		Testing position	Segment	Length/mm	Average diameter/mm
Trunk	A	A_1_	A_0_-A_1_	900	50.1
First branch	B_1_	B_11_	B_11_-A_1_	445	27.2
		B_12_	B_12_-B_11_	445	26.1
		B_13_	B_13_-B_12_	445	22.0
		B_14_	B_14_-B_13_	445	17.2
	B_2_	B_21_	B_21_-A_1_	292	35.5
		B_22_	B_22_-B_21_	208	33.2
		B_23_	B_23_-B_22_	343	20.8
		B_24_	B_24_-B_23_	343	18.2
		B_25_	B_25_-B_24_	343	13.9
Second branch	C_1_	C_11_	C_11_-B_21_	507	17.2
		C_12_	C_12_-C_11_	507	14.3
		C_13_	C_13_-C_12_	507	11.0
	C_2_	C_21_	C_21_-B_22_	393	19.8
		C_22_	C_22_-C_21_	393	16.8
		C_23_	C_23_-C_22_	393	14.5

### 2.2 Method of testing the fundamental frequency and damping ratio

**Fig 2:**
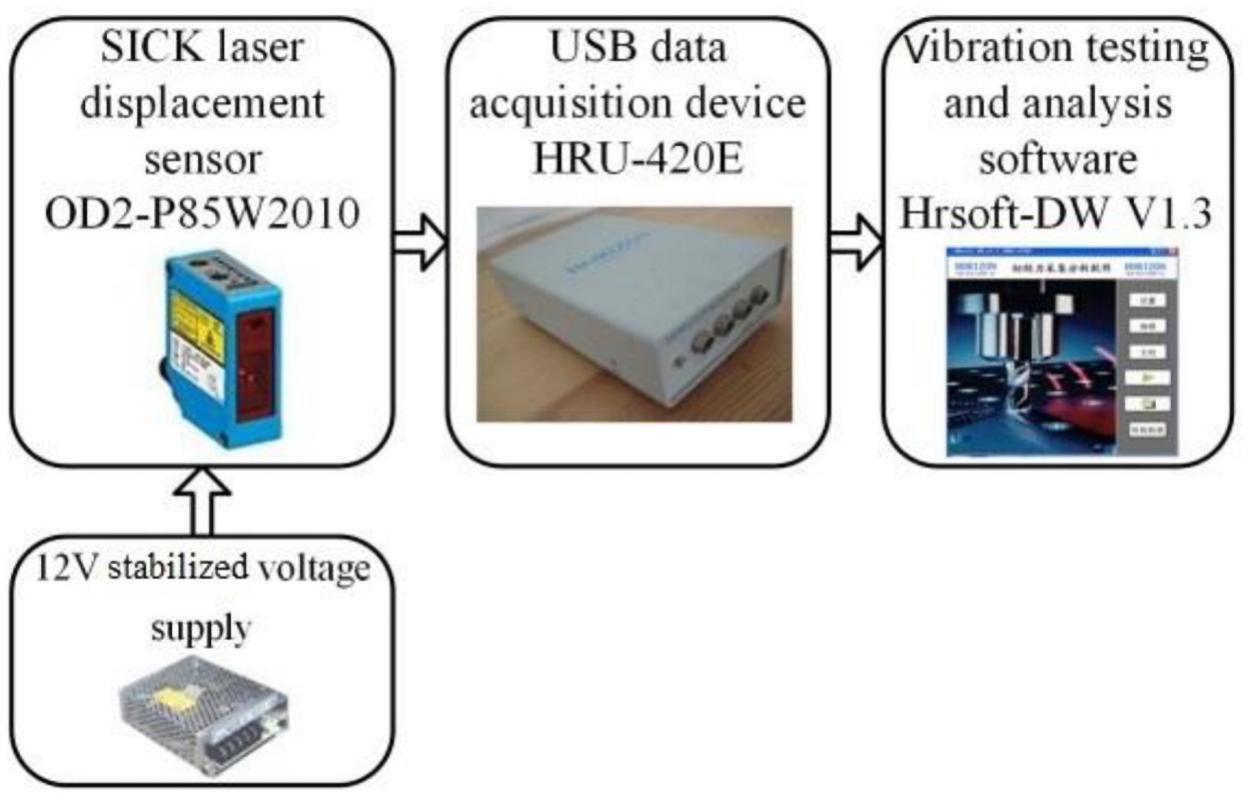
Equipment of testing fundamental frequency and damping ratio.

The fundamental frequency could be concluded by the pull-release test. As shown in Fig 2, testing equipment included a laser displacement sensor (OD-P85W20I0, SICK-Sensor Intelligence, Waldkirch, Germany), a 12V stabilized voltage supply (YU1220, Guangzhou Meanwell Electronic Product Co., Ltd. Guangzhou, China), a data acquisition device (HRU-420E, Shanghai Horizon Electronic Technology Co., Ltd. Shanghai, China), and a vibration testing and analysis software (HRsoft-DW V1.3, Shanghai Horizon Electronic Technology Co., Ltd. Shanghai, China).

A rope was tied on the trunk, then pulled and released to exert the step force on the tree specimen. The laser focused on a position 200 mm below the crotch node A_1_. The fundamental frequency (*ω*_0_, Hz) and damping ratio (*ξ*) could be calculated by the following equations, respectively:

$$\omega_{0}=\frac{n}{t_{n+1}-t_{1}}$$ (1)

$$\xi =\frac{\text{ln}(\frac{A_{1}}{A_{n+1}})}{n\cdot 2\pi }$$ (2)

**Fig 3:**
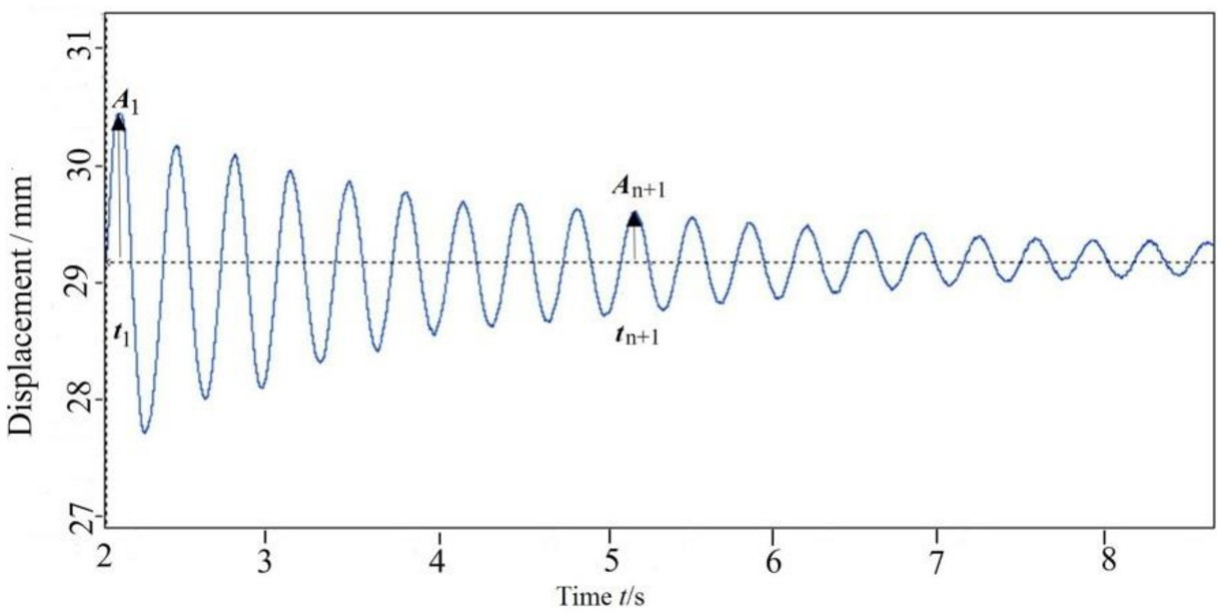
Displacement as a function of time under step force.

Where, *A*_1_ and *A*_*n*+1_ are the first and *n* + 1 peak values, *t*_1_ and *t*_*n*+1_ are the corresponding time to reach these two peaks in the displacement attenuation curve (Fig 3).

### 2.3 Method of testing the frequency spectrum characteristics and vibration responses

The frequency spectrum characteristics of tree specimen were excited by the impacting force produced by an impact hammer (LC-02A, Jiangsu Sinocera Piezotronics Inc, Yangzhou, China). By computing the power spectrum with the recording and analyzing software (CRAS V7.1, The First Test Software Engineering, Co., Ltd, Nanjing, China), the frequency at the peak and trough points could be indicated.

**Fig 4:**
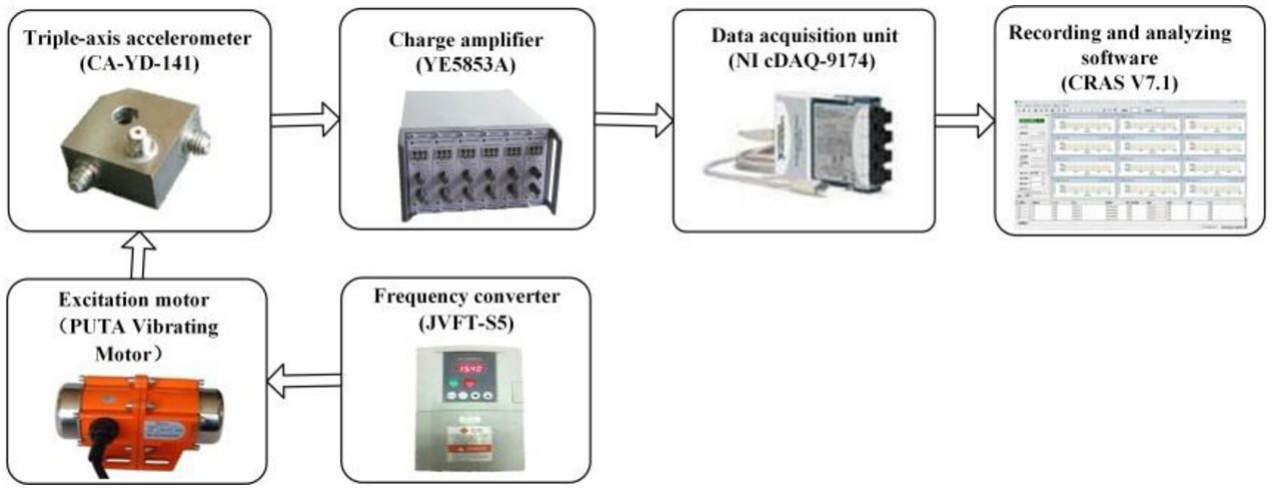
Schematic diagram of vibration response testing system.

As shown in Fig 4, the vibration response testing equipment consisted of a single-eccentric type harmonic excitation motor (Puta Vibrating Motor, Xin Jia Hong Technology Co., Ltd. Shenzhen, China), a frequency converter (JVFT-S5, Jinhui Instrumentation, Co., Ltd. Shenzhen, China), four triple-axis accelerometers (CA-YD-141, Jiangsu Sinocera Piezotronics Inc, Yangzhou, China), two charge amplifiers (YE5853A, Jiangsu Sinocera Piezotronics Inc, Yangzhou, China), and three data acquisition units (NI cDAQ-9174, The National Instrument Co., Ltd, USA).

The excitation motor was fixed at a position with 400 mm below the crotch node A_1_. The tree sample growing orientation was treated as the Z-direction. The X-direction was parallel to the connection line between the two central points of the motor and tree sample. As shown in Fig 1, Y-direction was vertical to these two directions. The triple-axis accelerometer could record the acceleration signals in three directions under the impact and harmonic excitations. In this paper, both the power spectrum amplitude and the acceleration response amplitude of each testing position were the resultant values of three directions.

Due to the limited number of accelerometers, only four testing positions could be tested in one test. During the experiment, when the treatments of four testing positions were completed, the accelerometers were moved to the next four testing positions until all positions were completed. The same treatment of each testing position was repeated three times and the test data was the average value of three measurements.

To describe the transmission characteristics of the dynamic response along the branches on the *Ginkgo biloba* tree, the dynamic acceleration transmission ratio (DATR) was defined using Eq (3).

$$k_{t(i)}=\frac{a_{i}}{a_{0}}$$ (3)

Where, *k*_*t*(*i*)_ is the DATR of the testing position *i*, *a*_*i*_ is the acceleration of the testing position *i*, *a*_0_ is the acceleration of the reference position. In this paper, the testing position A_1_ was regarded as the reference position.

## 3. Results and discussion

### 3.1 Frequency spectrum characteristics

For the *Ginkgo biloba* tree illustrated in Fig 1, through pull-release test, the fundamental frequency was 2.50 Hz and damping ratio was 0.06 by calculation of Eqs (1) and (2) when *n* was selected as 10 in Fig 3.

**Fig 5:**
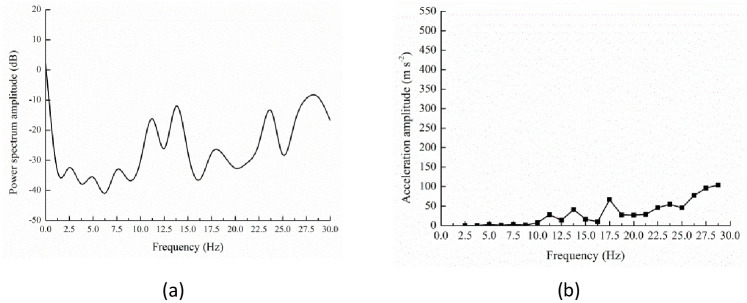
Power spectrum (a) and acceleration response (b) of testing position A_1_.

**Fig 6:**
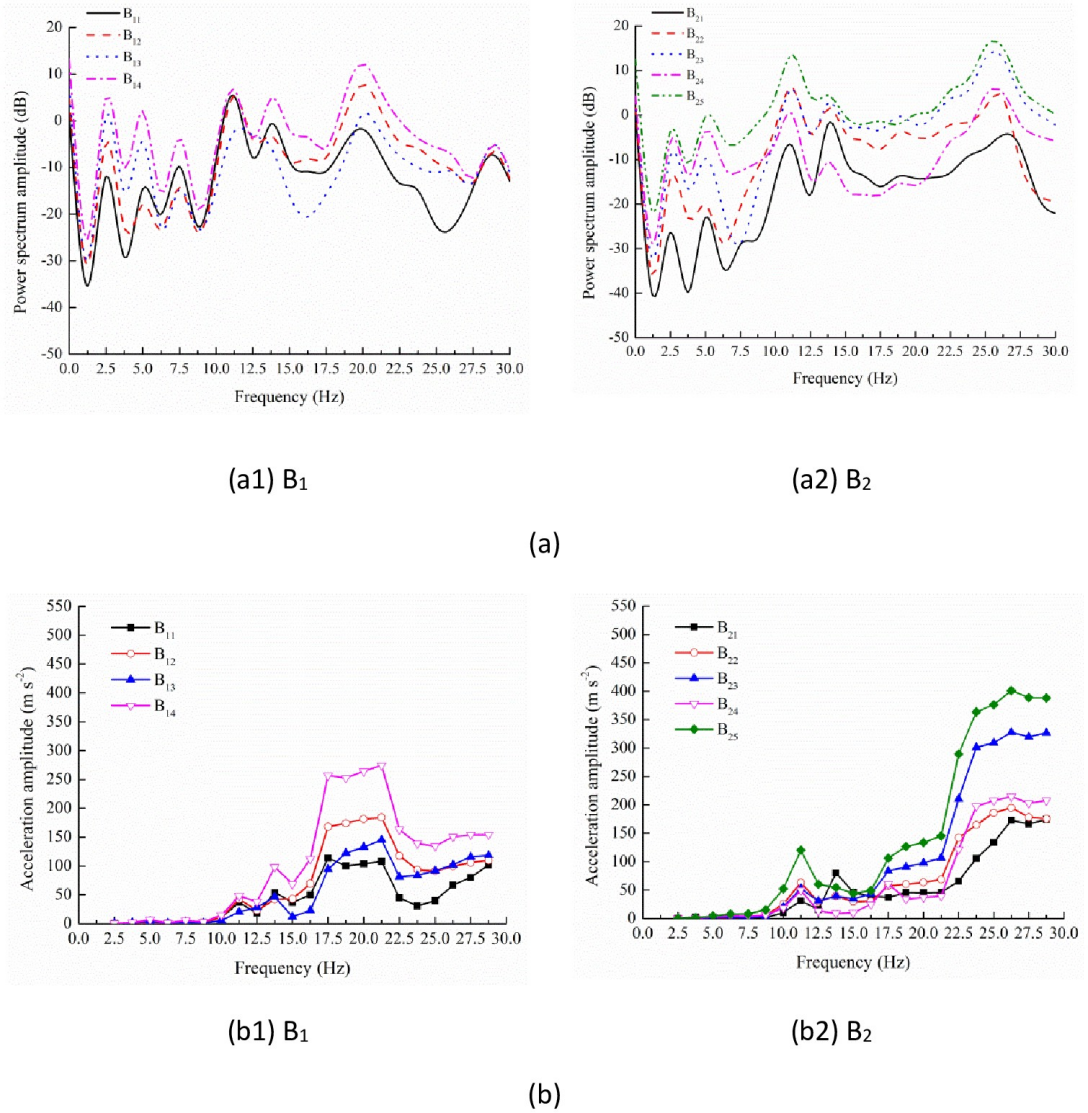
Power spectrum (a) and acceleration response (b) of testing positions on branches B_1_ and B_2_.

**Fig 7:**
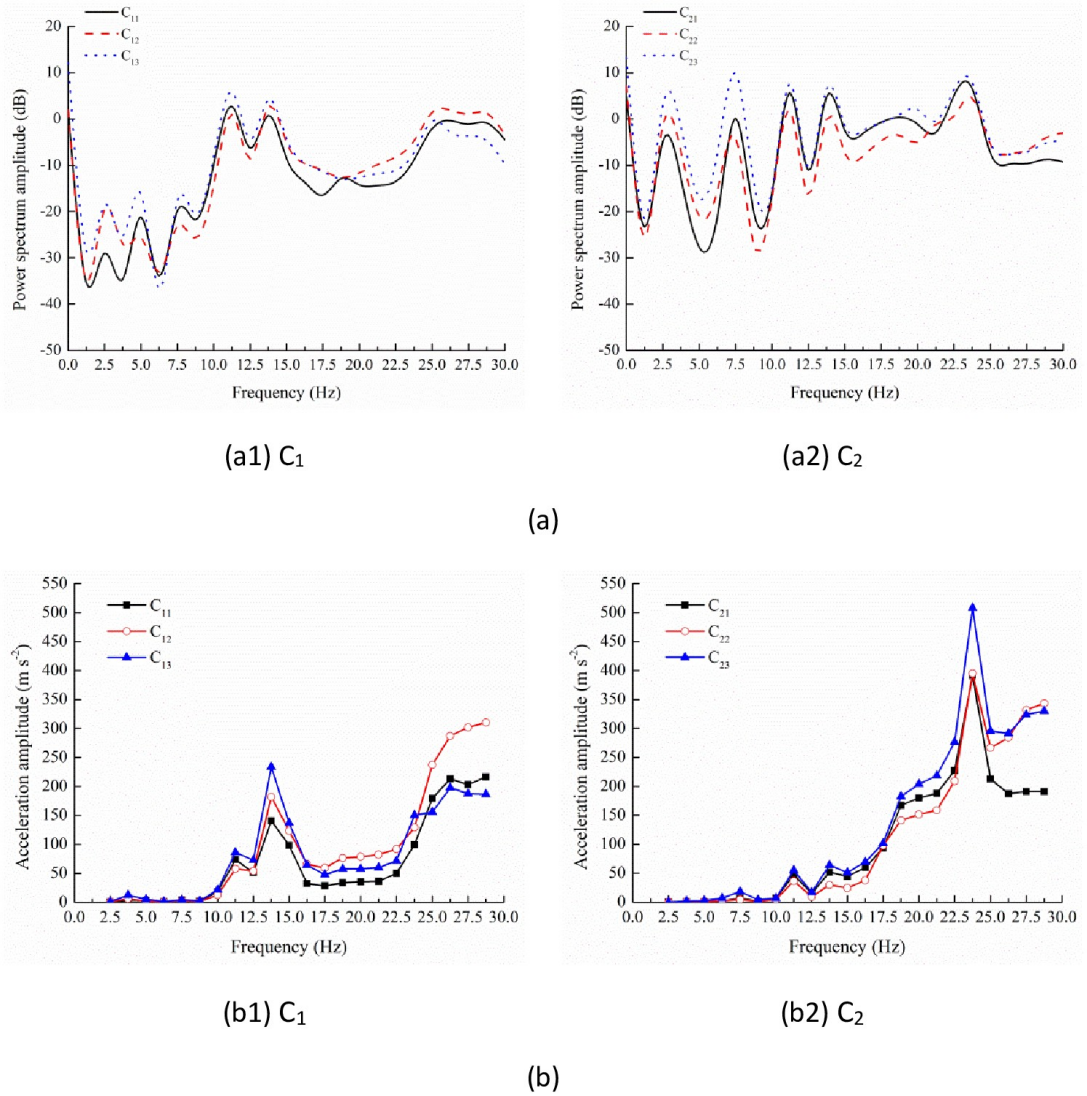
Power spectrum (a) and acceleration response (b) of testing positions on branches C_1_ and C_2_.

By the impact excitation test, the acceleration curve as a function of time could be attained. The power spectrum curves could be computed by the frequency spectrum treatment. The first peak point occurred at 2.50 Hz for all the power spectrum curves (Figs 5a, 6a and 7a), in accordance with the fundamental frequency by pull-release test. Since the appropriate excitation frequency was generally regarded as 10–30 Hz for the mechanical harvesting of fruits [33], the maximum analysis frequency was 30 Hz in the power spectrum.

At the testing position A_1_, small peaks continuously appeared at 5.00 and 7.50 Hz when the frequency was lower than 10.00 Hz. Between 10.00 and 20.00 Hz, the primary peaks could be found at 11.25, 13.75 and 17.50 Hz. At higher frequency, there were two peaks at 23.75 and 28.75 Hz (Fig 5a).

On the same branch, the power spectrum curves of different testing positions presented similar characteristics (Figs 6a and 7a). Unlike the testing position A_1_, 17.50 and 23.75 Hz weren’t the resonant frequencies for branches B_1_, B_2_ and C_1_. By contrast, on branch B_1_, there was another peak at 20.00 Hz. On branch B_2_, power spectrum curves also peaked at 26.25 Hz while there wasn’t a peak at 7.50 Hz. On branch C_1_, the lasting peak appeared at the frequency range 25.00–28.75 Hz. Nevertheless, the peaks only could be found at 7.50, 11.25, 13.75 and 23.75 Hz on branch C_2_. The identification of frequency peaks on different branches was summarized in Table 2. Therefore, among the frequency spectrum curves of different branches, there was consistency when the frequency was lower than 15.00 Hz. Nevertheless, at higher frequency, the frequency spectrum curves presented the peak points at different frequencies.

**Table 2:** Resonant frequencies of tree specimen.

Branch rank	Frequency(Hz)									
	I	II	III	IV	V	VI	VII	VIII	IX	X
A_1_	2.50	5.00	7.50	11.25	13.75	17.50		23.75		28.75
B_1_	2.50	5.00	7.50	11.25	13.75		20.00			28.75
B_2_	2.50	5.00		11.25	13.75				26.25	
C_1_	2.50	5.00	7.50	11.25	13.75					25.00–28.75
C_2_	2.50		7.50	11.25	13.75			23.75		

### 3.2 Vibration responses under the harmonic excitation

In order to completely analyze the relationship between the frequency spectrum characteristics and the vibration responses, tree specimen was excited at the frequency where the apparent and slight peak and trough points occurred in the power spectrum curves (Table 2, Figs 5a, 6a and 7a).

Testing position A_1_ located at the top of trunk and was the crotch node connecting branches B_1_ and B_2_. As shown in Fig 5b, the acceleration response was very weak at the I, II and III resonant frequency (Table 2). At the following resonant frequency of 11.25, 13.75 and 17.50 Hz, the acceleration amplitude reached the gradually increasing peaks 27.79, 40.71 and 66.65 m s^-2^. Due to the existence of the trough point in the power spectrum curve (Fig 5a), the value was only 9.72 m s^-2^ at 16.25 Hz. At the VIII resonant frequency 23.75 Hz, the acceleration response reached the maximal value 54.71 m s^-2^, which was smaller than the value at 17.50 Hz. When the excitation frequency was higher than 25.00 Hz, the acceleration amplitude increased obviously and attained the maximum 103.70 m s^-2^ at the X resonant frequency 28.75 Hz. It indicated that there was correspondence between the power spectrum characteristics and the acceleration responses.

For the branch B_1_ (Fig 6b1), the vibration responses were weak when the frequency was lower than 10.00 Hz. Then, at the IV and V resonant frequency 11.25 and 13.75 Hz, the maximal acceleration amplitudes of four testing positions attained 47.75 and 98.41 m s^-2^, respectively. At 17.50 Hz, the values increased sharply and then changed slightly with the frequency increasing to 21.25 Hz. At this frequency range, the average values reached 106.25, 177.00, 123.71 and 262.17 m s^-2^ for the testing positions B_11_, B_12_, B_13_ and B_14_. This was probably due to the existence of the resonant frequency 20.00 Hz (Table 2). Between 21.25 Hz and 25.00 Hz, the acceleration amplitudes decreased promptly and presented the apparent trough range. When the frequency was higher than 25.00 Hz, the values increased gradually. Although there was another resonant frequency 28.75 Hz, the vibration responses weren’t strengthened significantly again.

When the frequency was lower than 10.00 Hz, the vibration responses were also weak on branch B_2_ (Fig 6b2). At the IV resonant frequency 11.25 Hz, the vibration responses were strengthened and the value reached 120.32 m s^-2^ at the testing position B_25_. At the V resonant frequency 13.75 Hz, the acceleration amplitudes didn’t increase observably except for the value at the testing position B_21_. At the frequency range 15.00–21.25 Hz, the values presented increasing tendency but the increment was small. Nevertheless, when the frequency was higher than 21.25 Hz, the acceleration amplitudes of the five testing positions increased significantly and attained the maxima at the IX resonant frequency 26.25 Hz. The maxima were 2.76, 1.84, 2.06, 4.42 and 1.76 times larger than the values at 21.25 Hz, which was different from the characteristics on branch B_1_. At higher frequency, all the acceleration amplitudes decreased slightly and the value of the testing position B_25_ was still the largest.

For the branch C_1_ (Fig 7b1), the acceleration amplitudes of three testing positions were small at low frequency and increased to 58.03–86.29 m s^-2^ at the IV resonant frequency 11.25 Hz. At the V resonant frequency 13.75 Hz, the acceleration amplitudes reached 140.69, 181.89 and 233.63 m s^-2^ at the three testing positions C_11_, C_12_ and C_13_, which were much larger than the values at other testing positions. Then, the values decreased sharply and presented the trough range between 16.25 Hz and 22.50 Hz, which was in consistence with the power spectrum curve in Fig 7a1. However, when the frequency was higher than 22.50 Hz, the values presented significant increase. At 26.25 Hz, the peak values 213.30 and 197.93 m s^-2^ appeared at the testing positions C_11_ and C_13_. At higher frequency, all the acceleration amplitudes didn’t change a lot, which was similar to the values on branch B_2_.

Similarly to the testing position A_1_, branches B_1_, B_2_ and C_1_, the vibration responses weren’t strong until the frequency was higher than 10.00 Hz (Fig 7b2). The acceleration amplitudes attained small peaks at the IV and V resonant frequency 11.25 and 13.75 Hz. Then, the values increased gradually when the frequency was higher than 15.00 Hz. Especially at 18.75 Hz, the values increased by 79.08%, 44.75% and 79.71% than those at 17.50 Hz. At the VIII resonant frequency 23.75 Hz, the acceleration amplitudes reached 391.33, 395.15 and 508.15 m s^-2^, which were larger than the value of 363.65 m s^-2^ at the testing position B_25_. When the frequency was higher than 23.75 Hz, the acceleration amplitudes first decreased and then increased slightly. In addition, the values of the testing positions C_22_ and C_23_ were almost the same.

Consequently, there was a correspondence between the frequency spectrum characteristics and the vibration responses. Generally, the vibration responses were strengthened at the resonant frequency and attenuated at the frequency where the trough point appeared in the power spectrum curve. The vibration responses on the same branch presented the similar characteristics. Among different branches, the vibration responses were very weak when the frequency was lower than 10.00 Hz. At higher frequency, the vibration responses were strong but presented different characteristics.

### 3.3 Dynamic acceleration transmission characteristics

To reflect the transmission characteristics of the dynamic response, the DATRs of the testing positions on the tree specimen were calculated by Eq (3). The acceleration amplitudes under resonant frequencies 11.25, 13.75, 17.50, 20.00, 23.75 and 26.25 Hz were analyzed because they were identified as the most effective bands of excitation (Table 3).

**Table 3:** Obtained dynamic acceleration transmission ratio of different testing positions at resonant frequency.

Frequency/Hz	Testing position															
	A_1_	B_11_	B_12_	B_13_	B_14_	B_21_	B_22_	B_23_	B_24_	B_25_	C_11_	C_12_	C_13_	C_21_	C_22_	C_23_
11.25	1.00	1.33	1.53	0.73	1.72	1.13	2.28	1.90	1.78	4.33	2.65	2.09	3.10	1.70	1.32	1.98
13.75	1.00	1.33	1.03	1.13	2.42	1.97	0.98	0.97	0.22	1.34	3.46	4.47	5.74	1.27	0.73	1.57
17.50	1.00	1.70	2.52	1.42	3.86	0.56	0.86	1.25	0.92	1.59	0.42	0.89	0.72	1.40	1.47	1.52
20.00	1.00	3.85	6.76	4.94	9.84	1.69	2.36	3.64	1.39	4.98	1.32	2.92	2.15	6.70	5.63	7.59
23.75	1.00	0.56	1.71	1.53	2.54	1.93	3.01	5.51	3.61	6.65	1.81	2.36	2.75	7.15	7.22	9.29
26.25	1.00	0.87	1.29	1.32	1.95	2.24	2.52	4.24	2.78	5.19	2.76	3.72	2.56	2.42	3.68	3.77

On branch B_1_, most of the DATRs were larger than 1.00, which reflected that the dynamic response was mainly enhanced on this branch. At 20.00 Hz, the values of the four testing positions reached the maxima 3.85, 6.76, 4.94 and 9.84. At the same frequency, the maximal DATR always occurred at the testing position B_14_ which was located furthest away from the excitation point. Meanwhile, the minimum of the testing position B_14_ also reached 1.72. Therefore, the dynamic response was always the strongest on the top of branch B_1_.

On branch B_2_, in total, there were six DATRs smaller than 1.00 at 13.75 and 17.50 Hz. As the frequency increased, the value of the testing position B_21_ attained the maximum 2.24 at 26.25 Hz. Whereas, the values of the other four testing positions attained the maxima 3.01, 5.51, 3.61 and 6.65 at 23.75 Hz. At the same frequency, among the five testing positions, all the maximal DATRs occurred at the testing position B_25_ except for 1.97 of the testing position B_21_ at 13.75 Hz.

For the three testing positions on branch C_1_, the DATRs were only 0.42, 0.8 and 0.72 at 17.50 Hz. At the same time, the value of the junction point B_21_ was also smaller than 1.00. This exhibited that the dynamic response was attenuated when it was transmitted from the testing position A_1_ to the testing position B_21_ and branch C_1_. The maximal DATRs 3.46, 4.47 and 5.74 occurred at 13.75 Hz for the three testing positions C_11_, C_12_ and C_13_. At 11.25, 13.75 and 23.75 Hz, the maximum among the three testing positions occurred at the testing position C_13_. By contrast, the maximum was found at the testing position C_12_ when the frequency was 17.50, 20.00 and 26.25 Hz.

For branch C_2_, the only one DATR smaller than 1.00 occurred at the testing position C_22_ when the frequency was 13.75 Hz. Meanwhile, at the junction points B_21_ and B_22_, the values were 1.97 and 0.98. This reflected that the dynamic response wasn’t always enhanced during the process of transmission. As the frequency increased, the DATRs of the three testing positions attained the maxima 7.15, 7.22 and 9.29 at 23.75 Hz. The maxima were much greater than the values on other branches except for 9.84 of the testing position B_14_ at 20.00 Hz. Similarly to branch B_1_, at the same frequency, the maximal DATR appeared at the testing position on the top of branch.

The transmission characteristics of the dynamic response were affected by the branch rank and the excitation frequency. Due to the distinction in the frequency spectrum, the strongest dynamic response of different branches appeared at different frequencies. Generally, at the same frequency, the strongest dynamic response could be tested at the testing position which was located furthest away from the excitation position.

## 4. Conclusions

Test with the impact excitation and pull-release test revealed the same fundamental frequency 2.50 Hz for the tree specimen. On the same branch, the frequency spectrum exhibited the similar characteristics. Among the frequency spectrum of different branches, there was consistency when the frequency was lower than 15.00 Hz but distinction at higher frequency.

Generally, the vibration responses were consistent with the frequency spectrum characteristics. The vibration response induced by the single-eccentric type harmonic excitation could be strengthened at the resonant frequency. Merely, the acceleration responses at low frequency were very weak. At higher frequency, the vibration responses were strong but presented different characteristics among different branches. The acceleration amplitude on the trunk was always the smallest.

On the same branch, the dynamic responses of the testing positions presented the similar characteristics. Generally, the acceleration amplitude increased gradually as the testing position was located away from the excitation point. Comparing different branches, the strongest dynamic response appeared at different frequencies. It indicated that in the practical mechanical harvesting of fruits, it was difficult to induce the strong vibration response of all the branches at the single frequency.

The morphology of trees mainly contains monopodial branching and sympodial branching. The *Ginkgo biloba* tree with two levels of branch was representative. In future research, trees with other shapes could be tested to verify if the results of this study were universal.

## Supporting information

S1 Data (DOCX)
