# Supplementary material for: Vibration responses characteristics of a Ginkgo biloba tree excited under harmonic excitation
Source: PLoS One. 2021 Aug 20;16(8):e0256492. doi: 10.1371/journal.pone.0256492 (PMC8378724; doi:10.1371/journal.pone.0256492)
Supplement: S1 Data — (DOCX) [file pone.0256492.s001.docx]

Table 1. Dimension parameters of a *Ginkgo biloba* tree illustrated in Fig.1

| Branch rank | | Testing position | Segment | Length/mm | Average diameter/mm |
| --- | --- | --- | --- | --- | --- |
| Trunk | A | A_1_ | A_0_-A_1_ | 900 | 50.1 |
| First branch | B_1_ | B_11_  B_12_  B_13_  B_14_ | B_11_-A_1_  B_12_-B_11_  B_13_-B_12_  B_14_-B_13_ | 445  445  445  445 | 27.2  26.1  22.0  17.2 |
|  | B_2_ | B_21_  B_22_  B_23_  B_24_  B_25_ | B_21_-A_1_  B_22_-B_21_  B_23_-B_22_  B_24_-B_23_  B_25_-B_24_ | 292  208  343  343  343 | 35.5  33.2  20.8  18.2  13.9 |
| Second branch | C_1_ | C_11_  C_12_  C_13_ | C_11_-B_21_  C_12_-C_11_  C_13_-C_12_ | 507  507  507 | 17.2  14.3  11.0 |
|  | C_2_ | C_21_  C_22_  C_23_ | C_21_-B_22_  C_22_-C_21_  C_23_-C_22_ | 393  393  393 | 19.8  16.8  14.5 |

Table 2. Resonant frequencies of tree specimen

| Branch rank | Frequency(Hz) | | | | | | | | | | |
| --- | --- | --- | --- | --- | --- | --- | --- | --- | --- | --- | --- |
|  | Ⅰ | Ⅱ | Ⅲ | Ⅳ | Ⅴ | Ⅵ | Ⅶ | Ⅷ | Ⅸ | Ⅹ | |
| A_1_ | 2.50 | 5.00 | 7.50 | 11.25 | 13.75 | 17.50 |  | 23.75 |  | 28.75 | |
| B_1_ | 2.50 | 5.00 | 7.50 | 11.25 | 13.75 |  | 20.00 |  |  | 28.75 | |
| B_2_ | 2.50 | 5.00 |  | 11.25 | 13.75 |  |  |  | 26.25 |  | |
| C_1_ | 2.50 | 5.00 | 7.50 | 11.25 | 13.75 |  |  |  |  | 25.00-28.75 | |
| C_2_ | 2.50 |  | 7.50 | 11.25 | 13.75 |  |  | 23.75 |  | |  |

Table 3. Obtained dynamic acceleration transmission ratio of different testing positions at resonant frequency

| Frequency/Hz | Testing position | | | | | | | | | | | | | | | |
| --- | --- | --- | --- | --- | --- | --- | --- | --- | --- | --- | --- | --- | --- | --- | --- | --- |
|  | A_1_ | B_11_ | B_12_ | B_13_ | B_14_ | B_21_ | B_22_ | B_23_ | B_24_ | B_25_ | C_11_ | C_12_ | C_13_ | C_21_ | C_22_ | C_23_ |
| 11.25 | 1.00 | 1.33 | 1.53 | 0.73 | 1.72 | 1.13 | 2.28 | 1.90 | 1.78 | 4.33 | 2.65 | 2.09 | 3.10 | 1.70 | 1.32 | 1.98 |
| 13.75 | 1.00 | 1.33 | 1.03 | 1.13 | 2.42 | 1.97 | 0.98 | 0.97 | 0.22 | 1.34 | 3.46 | 4.47 | 5.74 | 1.27 | 0.73 | 1.57 |
| 17.50 | 1.00 | 1.70 | 2.52 | 1.42 | 3.86 | 0.56 | 0.86 | 1.25 | 0.92 | 1.59 | 0.42 | 0.89 | 0.72 | 1.40 | 1.47 | 1.52 |
| 20.00 | 1.00 | 3.85 | 6.76 | 4.94 | 9.84 | 1.69 | 2.36 | 3.64 | 1.39 | 4.98 | 1.32 | 2.92 | 2.15 | 6.70 | 5.63 | 7.59 |
| 23.75 | 1.00 | 0.56 | 1.71 | 1.53 | 2.54 | 1.93 | 3.01 | 5.51 | 3.61 | 6.65 | 1.81 | 2.36 | 2.75 | 7.15 | 7.22 | 9.29 |
| 26.25 | 1.00 | 0.87 | 1.29 | 1.32 | 1.95 | 2.24 | 2.52 | 4.24 | 2.78 | 5.19 | 2.76 | 3.72 | 2.56 | 2.42 | 3.68 | 3.77 |
